# As We Age: Listening to the Voices of LGBTQ Older Adults

**DOI:** 10.1093/geroni/igaa057.993

**Published:** 2020-12-16

**Authors:** Cory Cummings, Jennifer Dunkle, Bianca Mayes, Carolyn Bradley, Porsha Hall

**Affiliations:** 1 Monmouth University, West Long Branch, New Jersey, United States; 2 Stockton University, Atlantic City, New Jersey, United States; 3 Garden State Equality, Asbury Park, New Jersey, United States; 4 Monmouth University School of Social Work, West Long Branch, New Jersey, United States; 5 SAGE (Advocacy and Services for LGBT Elders), New York, New York, United States

## Abstract

LGBTQ older adults present a range of biopsychosocial needs and life experiences that may differ from the general population of older adults. Researchers have broken LGBTQ older adults into three age brackets: the Invisible Generation born before the 1920s; the Silent Generation born in the 1930s and 1940s; and the Pride Generation born in the 1950s and 1960s (Fredriksen-Goldsen, 2016). Research is emerging on health disparities and is fueling calls for inclusive services for this population. This paper session reports on the work of a research collaborative between social work and public health (two universities and a statewide advocacy organization). A qualitative study, designed as phase one a statewide need assessment, engaged ten focus groups (N=48 participants) throughout a mid-Atlantic state. Study aims were to better understand the experiences and perceptions of LGBTQ older adults now and expectations and plans for care as they age. Findings included (1) emphasis on the nuance of connection as an ageing LGBTQ adult; (2) expectations for quality of services; (3) realities of planning for future living arrangements; and (4) two sides of advocacy, as both a personal responsibility and a call for allyship. Recommendations will be made on how attendees can: evaluate agency policies and procedures to create safe spaces and inclusive services, engage in needs assessments of older LGBTQ+ adults in their own communities, and advocate at the State and Federal levels to strengthen services in the aging network to better serve this group, with specific focus on the Older Americans Act.

